# Physician Perspectives on the Initial Diagnostic Strategy of Syncope in Older Patients Without Diagnostic Clues

**DOI:** 10.1111/anec.70155

**Published:** 2026-01-30

**Authors:** Stephanie Happ, Satish R. Raj, Derek Chew, Robert Sheldon

**Affiliations:** ^1^ Department of Cardiac Sciences, Libin Cardiovascular Institute University of Calgary Calgary Alberta Canada

**Keywords:** implantable cardiac monitor, investigation strategy, physician preference, survey, syncope, tilt table test

## Abstract

**Background:**

Syncope in adults at least 50 years old without structural or electrical heart disease has numerous potential causes, and uncertainty persists in up to 41% of patients. Guideline‐directed investigations include implantable cardiac monitoring (ICM) and tilt table testing (TTT). Physician preferences about which to perform first are unknown. We aimed to understand physician opinions on whether to first utilize ICM or TTT to investigate syncope in older patients without electrical or structural heart disease.

**Methods:**

Physicians assessing syncope patients completed an online survey about diagnostic strategy, test availability, and values about test accuracy and clinical consequences.

**Results:**

Seventy‐one physicians completed the survey; 77% were cardiac electrophysiologists. Most respondents (62%) felt the optimum first test depended on the clinical scenario, 30% preferred an ICM, and 8% preferred a TTT. Tests were widely available: both tests were present in 76% of sites, TTT alone in 3%, ICM alone in 18%, and neither in 3%. TTT was preferred in Europe (75%) while ICM was preferred in North America (85%; *p* = 0.024). Concerns about missed diagnoses were expressed about TTT by 61% of physicians and only 19% for ICM. Following a negative first test, physicians were more likely to recommend an ICM (39%) than a TTT (11%), and more likely to watchfully wait after a negative ICM (45%) than a negative TTT (11%; *p* < 0.001).

**Conclusion:**

International equipoise exists about whether to pursue strategies of first conducting a TTT or implanting an ICM, although opinions differ between physicians practicing in Europe and North America.

**Trial Registration:**

ClinicalTrials.gov identifier: NCT05776810

## Graphical abstract

Physicians value timeliness and accuracy in choosing implantable cardiac monitoring (ICM) or tilt table testing (TTT) to investigate syncope in older patients without clues or heart disease. Europeans preferred 75% while North Americans preferred ICM 85%. 61% expressed concerns about missed diagnoses with TTT and only 19% for ICM.

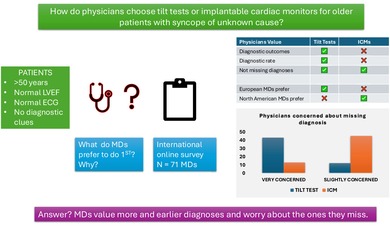

**Abbreviations**
ICM: implantable cardiac monitor
IQR: interquartile range
REDCap: Research Electronic Data Capture
TTT: tilt table test

Syncope is a common clinical concern that presents notable diagnostic challenges in the older adult population. Its incidence increases with advancing age, and older individuals often present to emergency departments with syncope‐related events, frequently necessitating acute care admissions. These presentations increase healthcare utilization and costs (Sandhu et al. 2016, 2018), and diagnostic evaluations are often inconclusive. Moreover, there remains a lack of consensus on the optimal diagnostic strategy (Shen et al. 2017; Brignole et al. 2018).

Obtaining an informative diagnostic history from older adults is often challenging due to factors such as the absence or poor recall of autonomic symptoms (O'Dwyer et al. 2011). Even after a thorough initial evaluation, the etiology of syncope remains unexplained in up to 41% of older patients (Shen et al. 2017). Current clinical guidelines provide limited direction regarding the optimal first diagnostic test. The 2017 American College of Cardiology/American Heart Association/Heart Rhythm Society guidelines (Shen et al. 2017), along with the 2018 European Society of Cardiology guidelines (Brignole et al. 2018), have Class IIa recommendations for the use of tilt table testing (TTT) and implantable cardiac monitors (ICM). Neither guideline, however, addresses the preferred sequencing. This might contribute to inconsistent practices, suboptimal outcomes, and variable healthcare expenditures.

This multinational study surveyed physicians on their evaluation strategies for older adults presenting with syncope of unknown etiology, as well as the factors influencing their clinical decision‐making. The aim is to understand physician preferences and some of the factors that inform them.

## 1 Methods

### 1.1 Study Design and Participants

This study was approved by the University of Calgary Conjoint Health Research Ethics Board (REB20‐1248). The target population for this survey included physicians from any medical specialty involved in the assessment of patients presenting with syncope. The survey was administered via the REDCap (Research Electronic Data Capture) platform, hosted on a secure University of Calgary server (Vandenberk et al. 2021). It was disseminated globally through the Heart Rhythm Society online forums and the Latin American Heart Rhythm Society community forums. Additional participants were recruited via email distribution, which included an open link that could be shared with other interested clinicians. Prior to participation, all respondents provided their informed consent electronically. Responses submitted from June 16, 2024 to August 8, 2024, were included in the present analysis.

### 1.2 Survey Design

The survey was available in English and consisted of eight sections. It stressed that the survey explicitly pertained to decision making around the diagnostic strategy of patients aged ≥ 50 years with syncope of undetermined etiology after a thorough initial examination and electrocardiogram, and excluded patients with known electrical or structural heart disease. Section 1 collected participant demographic information and information on medical practice and country of residence. Section 2 collected general factors influencing test selection. Section 3 pertained to the influence of clinical practice guidelines. Section 4 inquired about preferred tests and local availability. Section 5 pertained to physician opinions about patient preferences. Section 6 asked about strategies after an initial negative test; Section 7 asked about the influence of cost. Section 8 explored opinions about the interaction of safety concerns and test strategy.

### 1.3 Statistical Analysis

Categorical variables are presented as number and percentage. As continuous variables showed a nonnormal distribution, these data are presented as median and interquartile range (IQR). Continuous parameters were compared between groups using the nonparametric Mann–Whitney *U* test, and categorical comparisons were made using Fisher's exact test. A *p* value less than 0.05 was considered significant. All statistical analyses were performed using SPSS version 23 (IBM Corporation, Armonk, NY). GraphPad Prism 10.4.1 (GraphPad Software Inc., San Diego, CA) was used for figure creation.

## 2 Results

### 2.1 Respondents

A total of 88 physicians completed the survey, but 17 responses (19%) were incomplete and excluded from the analysis, resulting in a final analytic sample of 71 respondents (Table 1). Among participants, 77% identified as cardiac electrophysiologists, 23% were female, and the mean age was 54 ± 11 years. Over half of respondents (54%) had been in clinical practice for more than 20 years. The majority were based in North America (66%), followed by Europe (23%), Central and South America (7%), and Oceania (1%). Fully 94% of respondents reported that they actively manage patients presenting with syncope.

**TABLE 1:** Characteristics of survey respondents.

Characteristics of survey respondents (*N* = 71)	
Medical specialty	
Cardiac electrophysiology (EP)	55 (77%)
Cardiology (not EP)	10 (14%)
General internal medicine	1 (1%)
Other	5 (7%)
Female sex	16 (23%)
Age	
Less than 40	10 (14%)
41–50	12 (17%)
51–60	25 (35%)
Greater than 60	18 (25%)
Prefer not to state	6 (8%)
Years in practice	
Less than 5 years	8 (11%)
5–10 years	6 (8%)
11–20 years	19 (27%)
Greater than 20 years	38 (54%)
Location of practice	
North America	47 (66%)
South/Central America	7 (10%)
Europe	16 (23%)
Oceania	1 (1%)
Actively treats patients with syncope	67 (94%)

### 2.2 Test Modality Availability

There was regional variation in access to TTT and ICM (Table 2). In Europe, all responding sites reported availability of both TTT and ICM. In North America, 72% of sites offered TTT, 98% provided ICM, and 72% offered both modalities. Notably, 26% of North American sites provided only ICM, and none provided TTT alone. In Oceania and Latin America, 75% of sites offered TTT, 62% provided ICM, and 50% had access to both. In these regions, 12.5% of sites offered only ICM, while 25% provided only TTT. Overall, 79% of sites had access to TTT, 94% offered ICM, and 76% provided both. Among these, 18% of sites offered only ICM, and 2.8% offered only TTT.

**TABLE 2:** Diagnostic test availability by region.

Location	Access to testing modalities for syncope NYD				
	Both (%)	TTT (%)	ICM (%)	Neither (%)	Total
Europe	16 (100)				16
North America	34 (72)		12 (26)	1 (2)	47
Oceania	1 (100)				1
South/Central America	2 (33)	2 (33)	1 (17)	1 (17)	6
Other	1 (100)				1
Grand total	54	2	13	2	71

### 2.3 Physician Preferences for Initial Diagnostic Test

Overall, 8% of respondents preferred TTT as the initial diagnostic approach, 30% preferred ICM, and 62% indicated that their decision would depend on the clinical scenario. The survey had explicitly stated that no diagnostic clues were present and that both the electrocardiogram and left ventricular ejection fraction were normal. Among electrophysiologists who had a clear preference, a significantly greater proportion of North Americans selected ICM as their preferred first test (89%; Table 3) versus their European counterparts (25%; *p* = 0.024, 3 × 2 Fisher's exact test). The proportion of respondents who were uncertain about their preferred first test did not differ significantly between North America and Europe (54% vs. 64%; *p* = 0.73).

**TABLE 3:** Physician specialty and geographic variation in preferred initial tests for syncope evaluation.

Medical specialty	Preference of initial test for syncope NYD			
	TTT (%)	ICM (%)	It depends (%)	Total
Cardiac electrophysiology	5 (9)	19 (35)	31 (56)	55
Europe	3	1	7	11
North America	2	16	21	39
Oceania		1		1
Other (please specify)			1	1
South/Central America		1	2	3
Cardiology (not EP)		1 (1)	9 (90)	10
Europe			1	1
North America		1	6	7
South/Central America			2	2
General internal medicine			1 (100)	1
Europe			1	1
Other (please specify)	1 (33)		2 (67)	3
Europe			2	2
North America	1			1
Other internal medicine		1 (50)	1 (50)	**2**
Europe			1	1
Grand total	6	21	44	71

### 2.4 Physician Demographics

The relationship between physician demographics and preferred initial investigation for syncope was explored. There was no significant association between respondent‐reported sex and test preference (ICM, tilt testing, or no preference; *p* = 0.58; Fisher's exact test). There was a non‐significant age difference, with younger physicians favoring ICM, whereas older respondents more frequently indicated no clear preference (*p* = 0.060, Fisher's exact test) when the median age of 54 years was used as a cutpoint.

### 2.5 Test Performance

**FIGURE 1:**
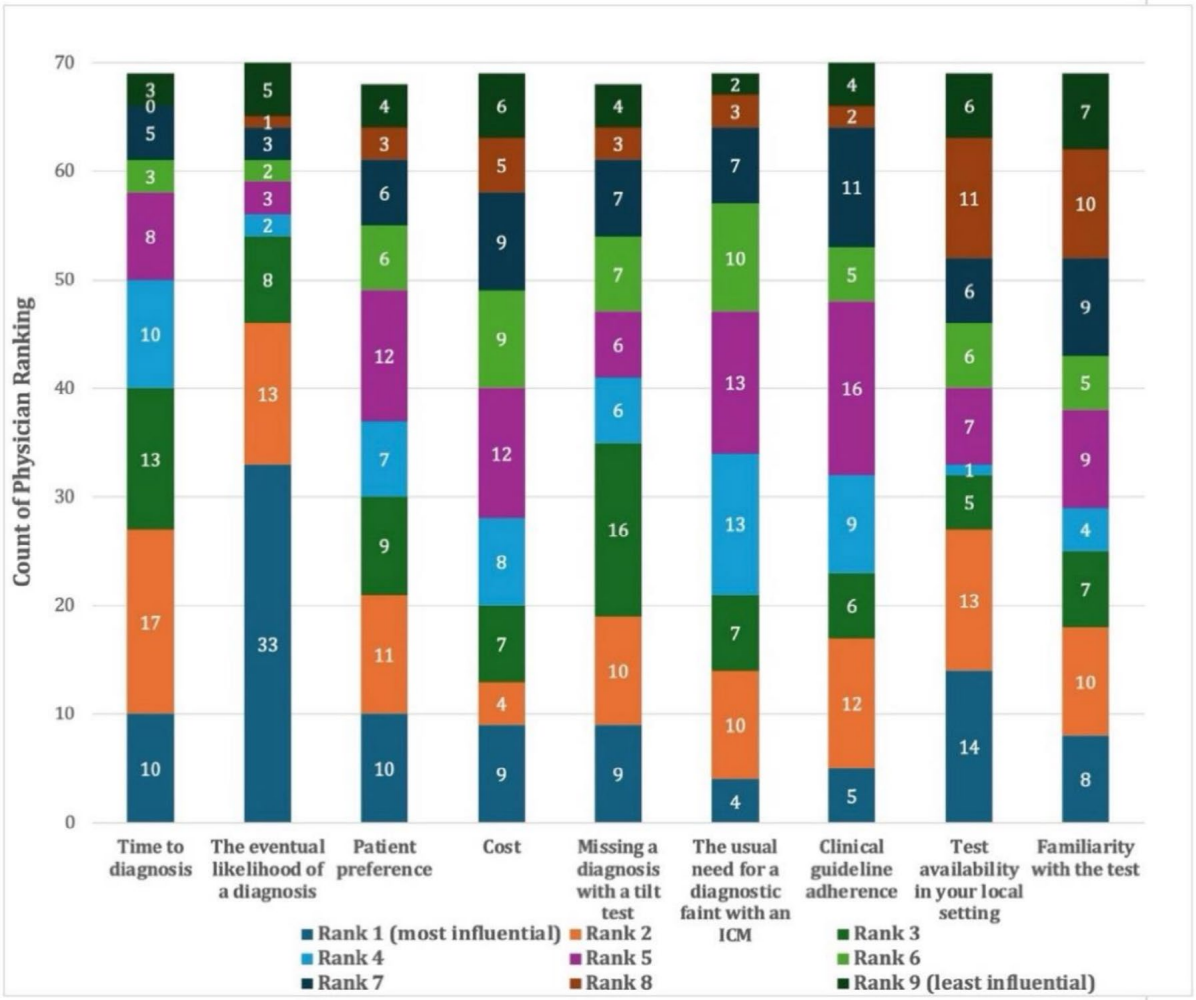
Physician prioritization of factors influencing test selection.

Physicians were asked to rank nine factors influencing their choice of diagnostic test, with #1 indicating the most important and #9 the least important (Figure 1). Not all respondents ranked every factor. The single most influential factor was the eventual likelihood of obtaining a diagnosis, ranked #1 by 33 of 71 respondents (46%). To simplify the analysis we combined rankings 1–3, 4–6, and 7–9 to comprise the top, middle, and third tertiles. The top tertile consisted of the eventual likelihood of obtaining a diagnosis, cited by 54 of 71 respondents (76%) as a top‐tier; time to diagnosis cited by 40 of 71 respondents (58%), missing a diagnosis with a TTT, cited by 35 of 68 respondents (51%). The lowest tertile included familiarity with the test (26/69, 41%), local test availability (23/68, 34%), and cost of testing (20/68, 29%).

### 2.6 Implications of Diagnostic Accuracy

**FIGURE 2:**
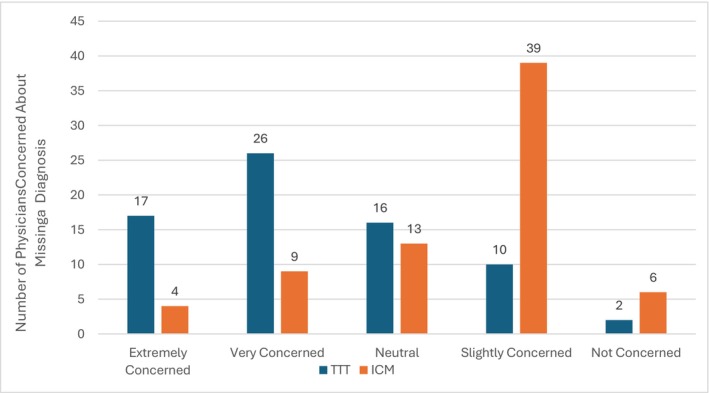
Physician concerns with diagnostic accuracy: HUTT versus ICM in syncope evaluation.

Physicians' perceptions of the diagnostic accuracy of TTT versus ICM revealed significant differences in concern regarding missed diagnoses (Table 4; Figure 2). Among respondents, 42 of 71 (59%) reported being “Extremely Concerned” or “Very Concerned” about missing a diagnosis with TTT, compared to only 13 of 71 (18%) for ICM. This difference was statistically significant (Fisher's exact test, 2 × 5 analysis, *p* = 0.005), suggesting that ICM is perceived as more diagnostically reliable or reassuring in the evaluation of syncope.

**TABLE 4:** Physician concerns with diagnostic accuracy: TTT versus ICM as an initial test.

Concern about missing a diagnosis?	TTT (%)	ICM (%)
Extremely concerned	24	6
Very concerned	37	13
Neutral	23	18
Slightly concerned	14	55
Not concerned	3	8

**FIGURE 3:**
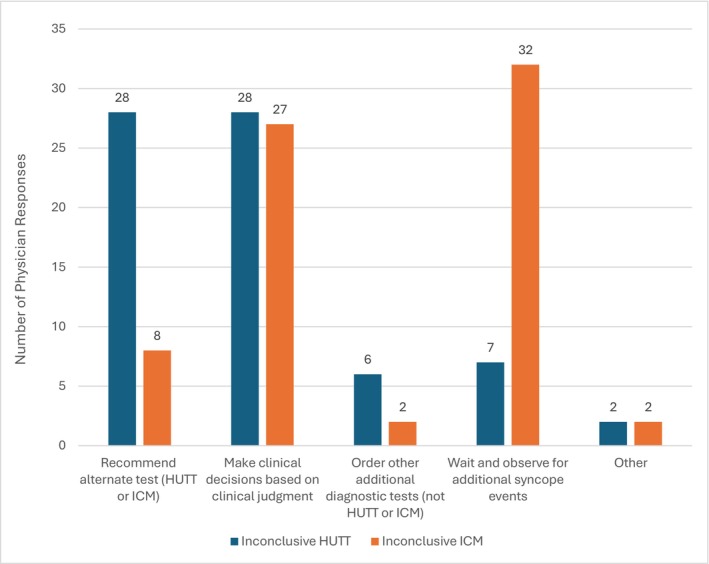
Physician actions following inconclusive HUTT and ICM results in syncope diagnosis.

When faced with inconclusive results (Figure 3), physicians were equally likely to rely on clinical judgment following either test (38% after TTT vs. 39% after ICM), but downstream decision‐making varied depending on which test was inconclusive. After a negative TTT, 28 of 71 (39%) respondents recommended proceeding with an ICM, and 7 (10%) recommended watchful waiting. In contrast, following an inconclusive ICM, only 8 respondents (11%) recommended a TTT, whereas 32 (45%) favored further watchful waiting. This significant asymmetry in decision patterns (Fisher's exact test, 2 × 5 analysis, *p* < 0.001) may reflect a perception of ICM as a more conclusive or terminal diagnostic modality.

### 2.7 Fear of Missed Diagnosis

When utilizing a TTT strategy, physicians were most concerned about missing diagnoses of heart block, supraventricular and ventricular tachycardia, sick sinus syndrome, vasovagal syncope, and epilepsy. In contrast, under an ICM‐based diagnostic approach, the primary concerns centered on missing hypotensive causes of syncope or arriving at no diagnosis at all (Table 5).

**TABLE 5:** Concerns of diagnoses missed with (top) TTT and (bottom) ICM.

	*n*	%
Missed diagnoses with TTT		
Heart block	13	35
SVT	5	14
Other (epilepsy, neuro mediated)	3	8
VT	2	5
Sick sinus syndrome	2	5
VVS	1	3
No physician response	11	30
Missed diagnoses with ICM		
No syncope recorded	4	25
Non‐cardiac cause	2	13
VVS	2	13
Orthostatic hypotension	2	13
No physician response	5	31

## 3 Discussion

### 3.1 Overview

There is a substantial difference in physician preferences for the initial diagnostic approach—TTT versus ICM—for older patients presenting with unexplained syncope, normal electrocardiograms, and preserved left ventricular ejection fraction. Overall, most physicians either expressed no strong preference. Among those with a preference, most physicians favored ICM as the first‐line investigation in this patient population. The preference for initial diagnostic strategies may be influenced by regional practice patterns, perceptions of test accuracy, and concerns about the clinical implications of missed or delayed diagnoses.

### 3.2 Indecision and Decision

One unexpected finding was how few physicians expressed a definitive preference for either of the initial test strategies. The survey had stipulated that the scenario involved older patients with unexplained syncope, a normal ECG, and preserved left ventricular ejection fraction—circumstances in which clinical uncertainty is often greatest. This lack of consensus may reflect the influence of other unmeasured factors such as patient frailty, risk of syncope‐related injury, or prior test experiences that we did not capture in this survey. Alternatively, it may speak to a broader sense of diagnostic equipoise or ambivalence among physicians when evidence is limited or conflicting.

### 3.3 Regional Differences

North American physicians were significantly more likely to prefer an ICM‐first strategy than their European counterparts. Interestingly, tilt testing was available at all European sites (100%) versus only 74% of North American sites, suggesting that availability might influence test preference. Alternatively, it is equally plausible that a greater cultural or clinical inclination toward TTT in Europe has driven its broader availability. This distinction may also reflect deeper trans‐Atlantic differences in diagnostic philosophy. European centers have led several influential, multicenter studies over the past three decades examining the diagnostic utility of TTT and ICM, as well as the physiological mechanisms of syncope (Forleo et al. 2013; Edvardsson et al. 2014, 2015; Raviele et al. 2000; Brignole 2007; Ungar et al. 2013; Brignole et al. 2000, 2001, 2006, 2014; Bartoletti et al. 2000; Moya et al. 2001; Solano et al. 2004; Brignole, Moya, et al. 2005; Brignole, Menozzi, et al. 2005). These academic traditions and evidence bases might have shaped physician perspectives.

### 3.4 Test Outcomes and Consequences May Drive Test Preference

Physicians' perceptions of diagnostic accuracy might influence the decision‐making process. The pooled top three priorities (Figure 1) in selecting a preferred test were “eventual likelihood of a diagnosis,” “time to diagnosis,” and “missing a diagnosis with a tilt test.” Fully 61% of physicians were either “Extremely Concerned” or “Very Concerned,” about not detecting significant cardiac conditions such as heart block (35%) and supraventricular tachycardia (14%) with a TTT. In contrast, only 19% of respondents were concerned about missing an important arrhythmic diagnosis with an ICM. When faced with inconclusive ICM results, 45% of physicians opted to “wait and observe for additional syncope events,” reflecting both a clinical tolerance for observational management and an awareness of the potential risks associated with missed diagnoses during this waiting period. Only a small proportion of respondents (8%) preferred tilt testing as the initial diagnostic tool. This limited preference likely reflects the electrophysiology community's greater familiarity with the diagnostic yield of ICM for identifying arrhythmic causes of syncope. ICM might be more aligned with physicians' risk–benefit thresholds in scenarios of diagnostic uncertainty.

While ICM are well‐suited for identifying arrhythmic causes of syncope (Edvardsson et al. 2011), they are less effective at diagnosing non‐cardiac and predominantly hypotensive etiologies. ICM also require a prolonged monitoring period, which may delay diagnosis. Despite these limitations, a high proportion of physicians opted to simply observe after an inconclusive ICM result. In contrast, many physicians would choose to escalate to ICM following a non‐diagnostic TTT. This divergence underscores the perceived diagnostic superiority of ICM and the influence of test‐specific limitations on downstream clinical decision‐making.

### 3.5 Most Important Factors

The most important factors for clinicians were focused on test accuracy and its implications: the eventual likelihood of obtaining a diagnosis, time to diagnosis, and missing a diagnosis with a TTT. This emphasis is highlighted by the thoughtfulness around what to do with a negative test. A negative TTT triggers a decision to implant an ICM, while a negative ICM triggers a decision to continue to watch.

### 3.6 Least Important Factors

The least important factors for clinicians were concerns about test familiarity, local availability, and testing costs (Figure 1). This is particularly noteworthy, given the significant differences in expenditures between tests (both upfront costs and follow‐up costs). Similarly, adherence to clinical practice guidelines was a secondary consideration for most respondents, although the guidelines do not recommend a preferred test to perform first (Shen et al. 2017; Brignole et al. 2018). These findings suggest that in real‐world decision‐making, physicians may prioritize patient‐specific factors and clinical context over more systemic considerations and general recommendations.

### 3.7 A Paradox Emerges

Physicians ranked the likelihood of diagnosis and time to diagnosis as the top priorities when selecting a test. Their preference for ICM over tilt testing, however, appears to contradict these values. For instance, an Italian tilt‐testing protocol yields a diagnosis in 63% of patients within a day (Forleo et al. 2013), while ICM provide a definitive diagnosis in about 30% over a 1 year time span (Edvardsson et al. 2011). This inconsistency may reflect a greater confidence in the diagnostic reliability of ICM or the perceived severity of conditions they detect. While tilt testing often identifies benign, treatable causes of syncope, ICM may uncover treatable albeit benign arrhythmias. Thus, despite being slower and less efficient, ICM may be preferred due to their perceived decisiveness and clinical impact.

### 3.8 Clinical Impact

This study highlights several considerations for knowledge translation into clinical practice. First, the high proportion of respondents who did not express a clear test preference might reflect the absence of head‐to‐head comparative data, underscoring the need for studies directly evaluating ICM‐first versus TTT‐first strategies. Second, the clear disconnect between the stated preference for the ICM‐first strategy and the data more supportive of a TTT‐first strategy highlights a need to better understand why clinical decision‐making diverges from current data. Third, an economic analysis of testing strategies to evaluate projected benefits and costs would provide valuable information to physicians and decision‐makers alike. Together, these findings highlight the need for better alignment between clinical practice, available evidence, and healthcare resource use.

The inability of many physicians to express a clear diagnostic preference may stem from the absence of direct comparative evidence between testing strategies. To address this, a randomized controlled trial is currently underway (NCT05776810). The Study To Understand Tilt Tests versus Extended Recordings (STUTTER, POST 10) is enrolling 120 patients aged 50 years or older who have experienced at least one syncopal episode in the past year, with no clear etiologic diagnosis and no clinically apparent structural or electrical heart disease. This pragmatic strategy trial compares HUTT and ICM as the initial diagnostic approach. The primary outcome is the establishment of a documented cause of syncope.

### 3.9 Limitations

This study has several limitations. First, the external validity is limited by the survey population itself. The sample population was limited by those we could access through privacy concerns of on‐line surveys. Participation was likely influenced by self‐selection bias. Recruitment was conducted through the Heart Rhythm Society and augmented by snowball sampling via professional networks, which may have favored engagement from certain subspecialty groups. It was also limited, as are all surveys, by those who took the time to complete the survey. This led to participants being mainly cardiac arrhythmia specialists, and not general cardiologists, internists, or emergency room physicians. However, European and North American syncope guideline committees are heavily influenced by arrhythmia specialists, suggesting the value of understanding the opinions and values of this group. An unknown proportion worked in syncope clinics, which are more common in Europe than in North America.

Second, we specified opinions about patients above 50 years of age. The reason is that younger patients generally have a rich variety of autonomic symptoms surrounding vasovagal syncope, and guidelines stipulate that a good history if clearly diagnostic suffices. Accordingly, we searched for opinions and values about investigating patients who were in an age group more likely to benefit from either a tilt test or an implantable cardiac monitor.

Third, the sample size was modest. The mean respondent age was 54 years, suggesting that the sample may not fully represent younger electrophysiologists or general cardiologists. We did not, however, observe a statistically significant association between age and test preference and did not inquire about specific age subgroups in our analysis. The small sample size prevented a deeper exploration of the effectiveness of guidelines on physician decision‐making.

## Author Contributions

All authors have contributed to each of the following: (1) conception and design of the study, and analysis and interpretation of data; (2) drafting of the manuscript or revising it critically for important intellectual content; and (3) final approval of the manuscript submitted.

## Funding

The authors have nothing to report.

## Ethics Statement

This study was approved by the University of Calgary Conjoint Health Research Ethics Board (REB20‐1248).

## Consent

All subjects provided informed written consent.

## Conflicts of Interest

The authors declare no conflicts of interest.

## Data Availability

The data that support the findings of this study are available on request from the corresponding author. The data are not publicly available due to privacy or ethical restrictions.
